# Endoplasmic Reticulum Stress and Lipid Metabolism

**DOI:** 10.1155/2012/257528

**Published:** 2012-06-19

**Authors:** Huiping Zhou, Kezhong Zhang, Sabina Janciauskiene, Xiaokun Li

**Affiliations:** ^1^Department of Microbiology and Immunology, School of Medicine, Virginia Commonwealth University, Richmond, VA 23298, USA; ^2^Hunter Holmes McGuire VA Medical Center, Richmond, VA 23298, USA; ^3^Center for Molecular Medicine and Genetics, Wayne State University School of Medicine, Detroit, MI 48201, USA; ^4^Department of Immunology and Microbiology, Wayne State University School of Medicine, Detroit, MI 48201, USA; ^5^Department of Respiratory Medicine, Hannover Medical School, Feodor Lynen Street 23, 30625 Hannover, Germany; ^6^College of Pharmacy, Wenzhou Medical College, Wenzhou, Zhejiang 325035, China

Endoplasmic reticulum (ER) is an elaborate cellular organelle essential for protein folding, calcium homeostasis, and lipid biosynthesis. Disruption of ER homeostasis imposes stress on the ER and subsequently leads to accumulation of unfolded or misfolded proteins in the ER lumen—a condition termed ER stress. In response to ER stress, a group of intracellular signaling pathways originated from the ER, collectively termed ER stress response, are activated to alter transcriptional and translational programs in the stressed cells. ER stress response has been linked to various human diseases associated with dyslipidemia, such as inflammatory diseases, obesity, diabetes, alcoholic and nonalcoholic liver diseases, and cardiovascular diseases. Understanding the impact of ER stress signaling pathways on lipid metabolism will provide important information for the prevention and treatment of these common human diseases in the modern world. 

The paper by C. Ji provided an updated overview of the potential mechanisms involved in alcohol-induced ER stress and organ injuries. The alcoholic injuries and roles of ER stress in major organs including liver, pancreas, brain, and heart were discussed. In addition, several important mechanisms underlying alcohol-induced ER stress were described in detail. 

The paper by S. Basseri and R. C. Austin provided an overview of recent findings related to ER stress and hepatic lipid metabolism. Liver is the central organ involved in lipid metabolism. Disruption of hepatic lipid metabolism has been linked to various metabolic diseases. Regulation of hepatic lipid metabolism and ER-stress signaling pathways was described. Furthermore, the therapeutic potential of targeting ER stress signaling pathways in dyslipidemia and obesity was discussed.

The paper by E. B. Thorp discussed the Unfolded-Protein-Response-(UPR)-induced apoptosis in ischemic cardiovascular disease. Ischemia is the major cause of heart failure which is secondary to dyslipidemia, atherosclerosis, and myocardial infarction. The ER stress signaling pathways in cardiomyocyte and role of ER stress in ischemia-induced apoptosis were discussed.

The paper by J. W. Brewer and S. Jackowski focused on the physiological UPR in the regulation of lipid synthesis and membrane biogenesis during the differentiation of B lymphocytes into antibody-secreting plasma cells. This paper described the current understanding of the relationship between the UPR, lipid biosynthesis, and organelle biogenesis in activated B cells. In particular, the authors provided up-to-date information regarding the roles and mechanisms of the UPR signaling pathways in regulating phosphatidylcholine synthesis and ER biosynthesis.

The paper by X. Zhang and K. Zhang discussed the links between ER stress, lipid droplet formation, and type II diabetes. The excessive deposition of lipid droplets in adipocytes, hepatocytes, and macrophages has been recognized as a feature of many metabolic diseases, including type II diabetes. Increasing evidence suggests that ER stress response regulates the lipid droplet formation that is associated with the pathogenesis of type II diabetes. This paper summarized the recent advances in understanding ER stress-associated mechanisms in lipid droplet formation and its involvement in type II diabetes.

The paper by B. S. Zha and H. Zhou provided an updated overview of ER stress response in lipid metabolism in adipocytes. Adipocytes are one of the major cell types involved in the pathogenesis of the metabolic syndrome. Recent advances in identifying the role of ER stress in regulating lipid metabolism in adipocytes and potential links among ER stress, inflammation, and autophagy were discussed.

*Huiping Zhou* *Kezhong Zhang* *Sabina Janciauskiene* *Xiaokun Li*

